# A novel microchannel-based device to capture and analyze circulating tumor cells (CTCs) of breast cancer

**DOI:** 10.3892/ijo.2014.2353

**Published:** 2014-03-21

**Authors:** REZA RIAHI, PRIYADARSHINI GOGOI, SAEDEH SEPEHRI, YI ZHOU, KALYAN HANDIQUE, JIM GODSEY, YIXIN WANG

**Affiliations:** 1 Ventana Medical Systems, Inc., A Member of the Roche Group, Tucson, AZ 85755;; 2 DeNovo Sciences, Plymouth, MI 48170, USA

**Keywords:** device, circulating tumor cells, breast cancer

## Abstract

Circulating tumor cells (CTCs) have been shown in many studies as a possible biomarker for metastasis and may be instrumental for the spread of the disease. Despite advances in CTC capturing technologies, the low frequency of CTCs in cancer patients and the heterogeneity of the CTCs have limited the wide application of the technology in clinic. In this study, we investigated a novel microfluidic technology that uses a size- and deformability-based capture system to characterize CTCs. This unique platform not only allows flexibility in the selection of antibody markers but also segregates the CTCs in their own chambers, thus, enabling morphological, immunological and genetic characterization of each CTC at the single cell level. In this study, different breast cancer cell lines including MCF7, MDA-MB-231 and SKBR3, as well as a panel of breast cancer biomarkers were used to test the device. The technology can capture a wide range of cells with high reproducibility. The capturing efficiency of the cells is greater than 80%. In addition, the background of leukocytes is minimized because individual cells are segregated in their own chambers. The device captured both epithelial cancer cells such as MCF7 and SKBR3 and mesenchymal cells such as MDA-MB-231. Immunostaining of the captured cells on the microchannel device suggests that a panel of breast cancer biomarkers can be used to further characterize differential expression of the captured cells.

## Introduction

Metastasis is the leading cause of death in patients diagnosed with cancer (1). Cancer metastasis occurs when tumor cells disassociate from the primary tumor, enter the circulation, and migrate to distant organs through the peripheral blood stream or lymphatic drainage. The development of metastases in patients is believed to result from tumor cells entering the circulation and migrating to distant organs (2,3). Circulating cells with the characteristics of tumor cells of epithelial origin or circulating tumor cells (CTCs) have been demonstrated to be present in breast, prostate and colon cancer patients’ blood and bone marrow (4–11). These cells have been shown not only in patients with metastatic diseases, but also in those whose tumors are apparently localized. Although CTCs are rare in patients, as a few as one cell per 100 million or 1 billion blood cells, molecular characterization of CTCs may provide a greater understanding of the disease metastases, help identify aggressive tumors, and enable therapeutic selection and monitoring of disease for patients undergoing treatment (4,5,7,9).

To develop technologies that identify and characterize CTCs and to establish the association of their presence with potential clinical significance have attracted tremendous interest in cancer research (6). A variety of technologies have been developed to improve detection and capture of CTCs from peripheral blood, which include immunomagnetic bead separation using monoclonal antibodies targeting epithelial cell-surface antigens, cell sorting using flow cytometry, filtration based size separation, density gradient centrifugation, microfluidic devices and fast-scan imaging (12–18). For example, CellSearch™ was the first rare cell technology that demonstrated its clinical validity in predicting progression-free survival and overall survival of metastatic cancer patients based on CTC enumeration (4,5,7,9). Despite advances in CTC capturing technologies, the low frequency of CTCs in cancer patients and the heterogeneity of the tumor and the CTCs have limited applications of the CTC technology in clinic. Current technologies for CTC detection suffer from extensive leukocyte contamination and dependency on either tumor specific or epithelial specific immune markers for the capture of the target cells, making it highly unlikely that one single perfect marker exists that will identify all the CTCs present in the same tumor and within the same patient (6,19). For example, the epithelial cell adhesion molecule (EpCAM) represents the current capturing antibody of choice for the majority of microfluidic devices that have been developed to capture CTCs. However, the use of EpCAM as the capturing antibody has been criticized. There is strong evidence from preclinical and clinical studies that a small population of the CTCs undergoes an epithelial to mesenchymal transition (EMT) and spread the tumor to distant organs (20). Relying on one capturing antibody is not the best strategy for CTC identification and the solution lies in multiplexing cancer biomarkers to identify as many heterogeneous cancer cells as possible to study their roles in cancer progression.

Furthermore, it is of great interest to go beyond cell enumeration and characterize the CTCs by assessing gene and protein markers on CTCs to gain insight into mechanisms of metastasis and best treatment modalities for patients (21,22). For example, breast cancer encompasses a group of highly heterogeneous diseases, which can be demonstrated at molecular, histopathologic and clinical levels. In no other cancer has there been so much research linking the role of various biomarkers to disease progression and patient outcome. Significant progress has been made over years in breast cancer detection and management, including annual mammographic screening, effective hormonal and chemotherapy therapies, and targeted therapies against estrogen receptor (ER) and HER2. With such progress, it becomes critically important to determine which patients are most likely to benefit from which therapies and in identifying subgroups of patients who have a more aggressive disease thus are at the highest risk for recurrence. For example, decision regarding the use of adjuvant therapies requires weighing the risk of recurrence against the potential benefit and side-effect of a treatment. Established clinical, pathologic features and biomarkers such as patient age, tumor size, nodal status, tumor grade, ER, progesterone receptor (PR), and HER2 status are used to estimate a patient’s risk for recurrence and to guide treatment options. However, these types of risk estimates remain imprecise for many patients, which lead to either over-treatment of some with unnecessarily toxic therapies, or to under-treatment of others who receive false assurances of a favorable prognosis. Attempts have been made to identify additional molecular markers that could predict disease progression and patient outcome more precisely (1,23–25). Studies on gene expression microarray have led to the discovery of distinct subtypes of breast carcinomas, each with unique phenotypes and clinical outcomes. Similar studies have shown that breast cancer can also be divided into 5 similar subgroups using immunohisto-chemical (IHC) analysis with a panel of protein markers (such as ER, PR, HER2, Ki67, PI3K and others) (23–25). To detect such molecular markers using a minimally-invasive test such as CTCs has a great potential for use in routine clinical practice to guide therapy choice for breast cancer patients.

In this study, we have developed a novel microfluidic technology that uses a size and deformability based capture system to characterize CTCs. The JETTA™ microfluidic chip contains a parallel network of fluidic channels which have about 56,320 capture chambers (26). Each chamber ensures that smaller blood cells such as red blood cells and most of the leukocytes escape while larger cancer cells get trapped and isolated in the chamber. Because the device captures cells using label free detection, it is wide open to using a variety of antibodies. In addition, since target cancer cells are segregated in their own chambers separate from leukocytes, it alleviates the problem of leukocyte contamination that is associated to most of current CTC technologies. Most importantly, the single cell capturing chamber has the potential to allow downstream molecular analysis such as polymerase chain reaction (PCR), fluorescence *in situ* hybridization (FISH) and IHC assays to be performed on the microfluidic device at the single cell level. This capability distinguishes the technology from all other available CTC technologies and provides tremendous hope for the field to go to the next stage of clinical validation of CTCs.

To validate this microfluidic technology, different breast cancer cells including MCF7, MDA-MB-231 and SKBR3, as well as a panel of breast cancer biomarkers were used to test the device (27). We found that the device captured cells in a range of 20–2,000 with high reproducibility. The capturing efficiency of the cells was greater than 80%. In addition, background leukocyte in the captured cell population is minimized. Furthermore, it captured both epithelial cancer cells such as MCF7 and SKBR3 and mesenchymal cells such as MDA-MB-231. Immunostaining of the captured cells on the microchannel device suggested that a panel of breast cancer biomarkers can be used to characterize differential expression of the captured cells.

## Materials and methods

### Microfluidic chip fabrication process

**Figure 1.**
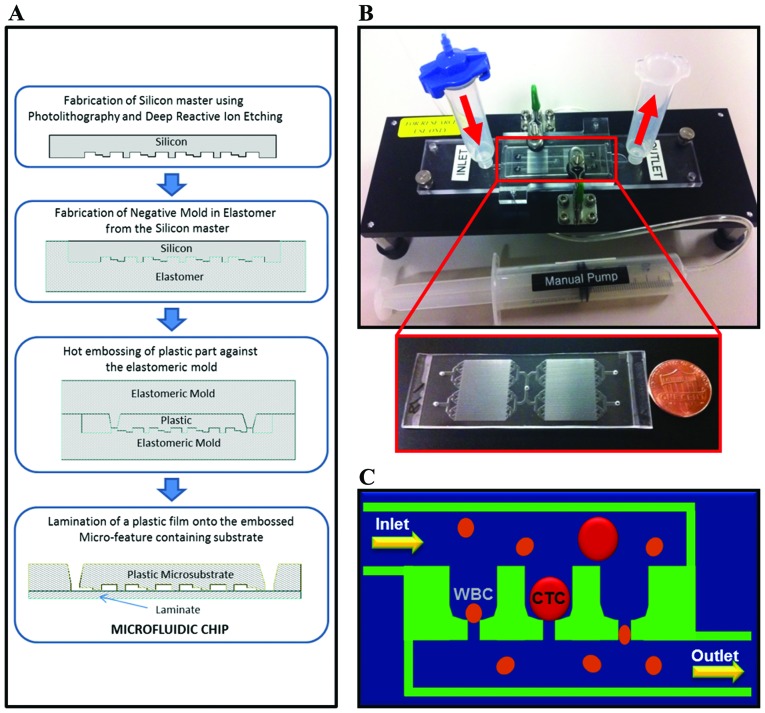
Schematic drawing of microchannel device and work flow. (A) The microfluidic chip fabrication process. (B) The microchannel device is illustrated. (C) The size-based filtration for CTC capturing.

The microfluidic chip fabrication begins with a silicon master device containing micro-features (Fig. 1A). The micro-features consist of a fluidic network (∼75 *μ*m deep) leading to multiple cell trapping chambers (20 × 25 × 30 *μ*m) with individual pore channels (∼10 × 8 *μ*m). This process uses standard micro-fabrication techniques (photo-lithography and deep reactive ion etching). From the master device, a soft elastomeric negative mold is created by pouring and curing against the silicon master. The final micro-substrate is created by hot embossing a plastic plate made of cyclic olefin polymer (COP) against the elastomeric negative mold. A thin plastic laminate containing pressure-sensitive adhesive is then laminated against the COP micro-substrate to create the final microfluidic chip. The microchannel device is illustrated in Fig. 1B and the size-based filtration for CTC capturing is described in Fig. 1C.

### Cell line and cell culture

Several breast cancer cell lines were used for microchannel device testing and in spiked-in experiments. Human mammary carcinoma cell lines MCF7 (ATCC HTB-22), MDA-MB-231 (ATCC HTB-26), and SKBR3 (ATCC HTB-30) were obtained from American Type Culture Collection (ATCC, Manassas, VA). MCF7 and MDA-MB-231 cells were cultured in DMEM medium with 10% deactivated fetal bovine serum (FBS) (Life Technologies, Carlsbad, CA) and 1% Pen Strep (Life Technologies). SKBR3 cells were cultured in McCoy’s 5A medium with 10% deactivated FBS (Life Technologies) and 1% Pen Strep (Life Technologies). The cultures were maintained at 37°C in a humidified atmosphere containing 5% CO^2^ (v/v). The cells were sub-cultivated every 4 days and the media was replaced every 48 h. Sub-confluent monolayers were dissociated using 0.25% trypsin solution (Thermo Scientific, Waltham, MA).

### Sample preparation and cell capture

Peripheral blood samples were obtained from healthy donors using CellSave tubes (Veridex, Raritan, NJ) with written informed consent (Boca Biolistics, Coconut Creek, FL). A known amount of cells diluted in cell culture medium were introduced to 2 ml of 1X phosphate-buffered saline (PBS) or 2 ml of normal blood sample and prefixed in 2 ml 0.8% paraformaldehyde (PFA) using a tube rocker for 10 min incubation. Prior to sample loading, the microfluidic device was coated with priming buffer consisting of 1X PBS, ethylene-diamine-tetraacetic acid (EDTA), and 1.0% bovine serum albumin (BSA) to coat microchannels and remove bubbles. The prepared sample was then added into the inlet reservoir, followed with loading into the microfluidic device at approximately 1 ml/min volumetric flow rate. Cancer cells owing to their bigger size compared to blood cells were captured by micro-chambers and the remaining solution containing red blood cells and most of the leukocytes is collected by the outlet reservoir after passing through pore chambers. A background level of larger leukocytes such as monocytes are also trapped by the micro-chambers but are distinguished by their surface markers in the subsequent analysis.

### Immunofluorescence staining of CTCs

After being captured in the microchannel device, prefixed cells were fixed using 4.0% PFA for 10 min at room temperature. Permeabilization was then achieved by 0.1% Triton X-100 (Sigma-Aldrich, St. Louis, MO) and 1.0% BSA for 10 min at room temperature. After blocking with 5% Goat Serum (Life Technologies) for 25 min, the cells were incubated for 50 min with mouse monoclonal antibodies. AlexaFlour 488 conjugated antibodies against either vimentin (Santa Cruz Biotechnology, Santa Cruz, CA) or E-cadherin (BD Biosciences, San Diego, CA) were diluted 1:100 for staining. Monoclonal IgG1 primary antibodies against HER-2 (BioLegend, San Diego, CA), ER (BD Biosciences), PI3K (Abcam, Cambridge, MA), and PanCK (Sigma-Aldrich) were diluted 1:200. All primary antibodies were then detected by anti-mouse AlexaFluor 488 secondary IgG1 antibody with 30 min incubation. The antibody against leukocyte common antigen, CD45 (mouse IgG2a) (AbD Serotec, Oxford, UK) was diluted 1:200 and used as a marker for background leukocytes. CD45 was then detected by anti-mouse AlexaFluor 594 secondary IgG2 antibody (1:500 dilution) (Life Technologies). Nuclei were counterstained with 1.0 *μ*g/ml Hoechst-33342 (Life Technologies) for 5 min after secondary antibody incubation.

### Microscope imaging, enumeration and analysis of CTCs

Cells were monitored using an inverted fluorescence microscope TE2000-U (Nikon, Tokyo, Japan). Bright-field and fluorescence images and time lapse videos were captured using a HQ2 CCD camera (Photometrics, Tucson, AZ). All images were taken with the same exposure time and conditions in order to compare the relative fluorescence intensity. Data collection and imaging analysis were performed using the NIH ImageJ software. CTC enumeration following antibody labeling was performed manually. PanCK^+^/CD45^−^ nucleated cells were identified as CTCs. Positive and negative controls for antibody performance and staining were included in each experiment. Each experiment was performed in triplicates and results are expressed as means ± SE for each set of experiments.

## Results

### Enumeration and capture efficiency of cells

**Figure 2.**
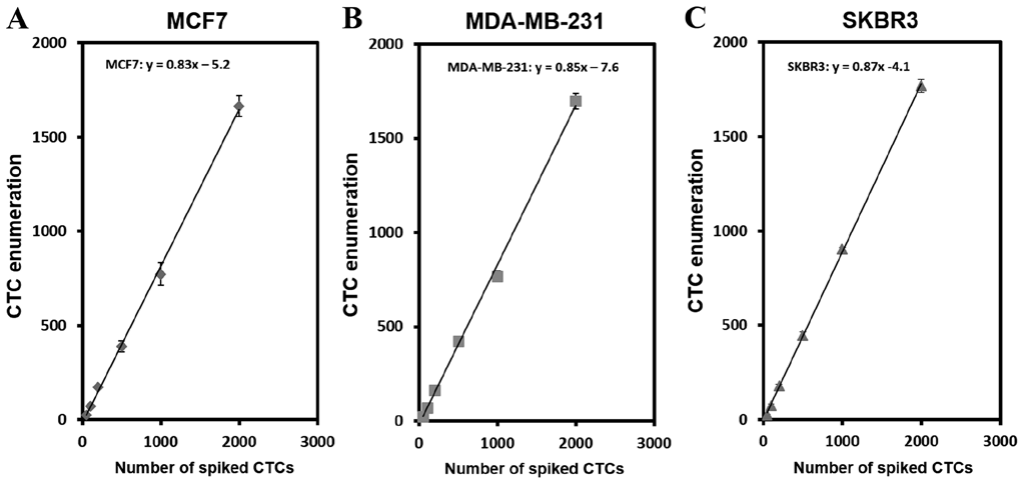
Capturing efficiency of breast cancer cells in PBS. The capture efficiency of cells using different cell lines in 1X PBS is used to show the performance of the microchannel device. (A) MCF7 cells, (B) MDA-MB-231 cells, (C) SKBR3 cell.

To test the performance of the microchannel device, we first determined the capture efficiency of cells using cell lines in 1X PBS (Fig. 2). As shown in Fig. 2A, different number of MCF7 cells, ranging from 20 to 2,000 cells per 2 ml 1X PBS, were analyzed. The average capture efficiency is 83%. Similar experiments have been also performed with MDA-MB-231 and SKBR3 cell lines yielding averaged capture efficiency of 85% and 87%, respectively (Fig. 2B and C). Table I shows the capture efficiency of the device at each number of spiked cells. The efficiency of cell capturing ranged between 75–83% for MCF7, 77–85% for MDA-MB-231 and 78-89% for SKBR3. Coefficient of variance obtained by three independent experiments (n=3) varied between 2.5 to 6.7 suggesting high reproducibility of cell capturing with this device.

### Enumeration and capture efficiency of spike-in cells

**Figure 3.**
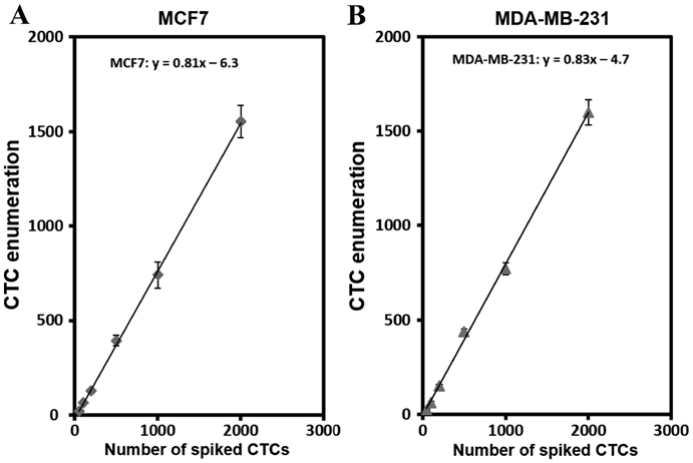
Capturing efficiency of breast cancer cells spiked in healthy donor blood. To assess cell capture efficiency under physiological conditions, a series of spike-in experiments in which certain number of breast cancer cells including (A) MCF7 (epithelial), (B) MDA-MB-231 (mesenchymal) were spiked into peripheral blood samples from healthy donors.

To assess cell capture efficiency under physiological conditions, we performed a series of spike-in experiments in which certain number of breast cancer cells including MCF7 (epithelial) and MDA-MB-231 (mesenchymal) were spiked into peripheral blood samples from healthy donors. As shown in Fig. 3, the average cell capturing efficiency in the spike-in samples was 81% and 83% for MCF7 and MDA-MB-231 cells, respectively. The results showed that the capture efficiency of two cell lines was quite comparable ranging from 74–82% for MCF7 and 75–82% for MDA-MB-231 depending upon number of spiked cells (Table II). Low coefficient of variance (1.0–6.5) indicated high reproducibility of the results (n=3). This data further demonstrated that the capture efficiency and experimental reproducibility for each cell spiking number are consistent with the results we observed for the cells in PBS buffer. We have tested 20 MCF7 cells spiked into 2 ml blood samples, the average cell capturing efficiency was 84% with the standard deviation of 11.9% (n=5). In addition, spiked-in samples with 5 MCF7 cells yielded 4 or 5 cells in multiple tests although the accuracy of cell counts is difficult to achieve at this level (data not shown). Collectively, high capture efficiency and reproducibility were evident with the device in both the cell lines and the spike-in samples.

### Molecular characteristics of cells

To examine the ability of the microchannel device to characterize the captured cells with molecular markers, we performed a series of immunostaining experiments to analyze the expression of several breast cancer epithelial or mesenchymal-specific biomarkers. MCF7, MDA-MB-231 and SKBR3 cells were used in the experiments.

**Figure 4.**
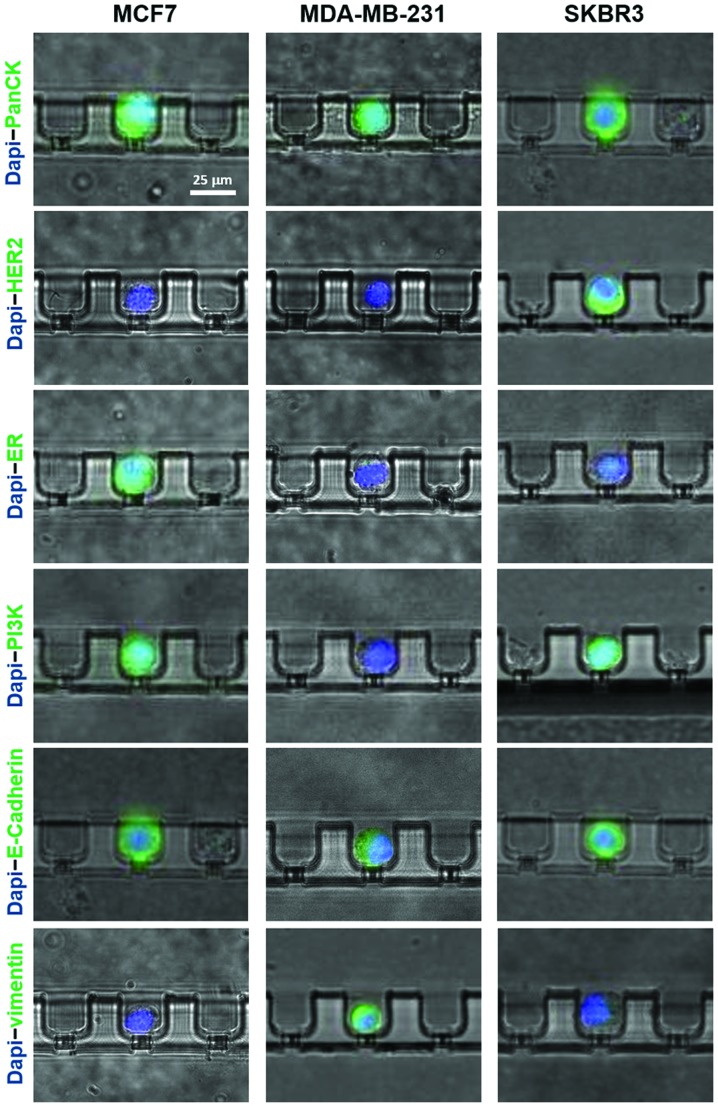
Immunostaining of captured cells in PBS. For each cell line (columns), 100 cells were first spiked into 2 ml 1X PBS and then stained with either PanCK, HER-2, ER, PI3K, E-cadherin or vimentin after being captured by microchambers (rows). The cell nuclei were also stained by 1.0 *μ*m/g Hoechst-33342 in all cases.

For each cell line, 100 cells were first spiked into 2 ml 1X PBS and then stained with either PanCK, HER-2, ER, PI3K, E-cadherin or vimentin after being captured by microchambers (Fig. 4) The cell nuclei were also stained by 1.0 *μ*m/g Hoechst-33342 in all cases. Our observation revealed positive staining of PanCK in all three cell lines. HER-2 was only expressed in SKBR3 cells, but not MCF7 cells. ER was only expressed in MCF7 cells, but not SKBR3 cells. In addition, both epithelial cells, MCF7 and SKBR3 were PI3K and E-cadherin positive, but vimentin negative. Compared to the epithelial cells, MDA-MB-231 was shown to be HER2, ER, PI3K negative while expressing a low level of E-cadherin and high level of vimentin, a mesenchymal cell-specific marker.

**Figure 5.**
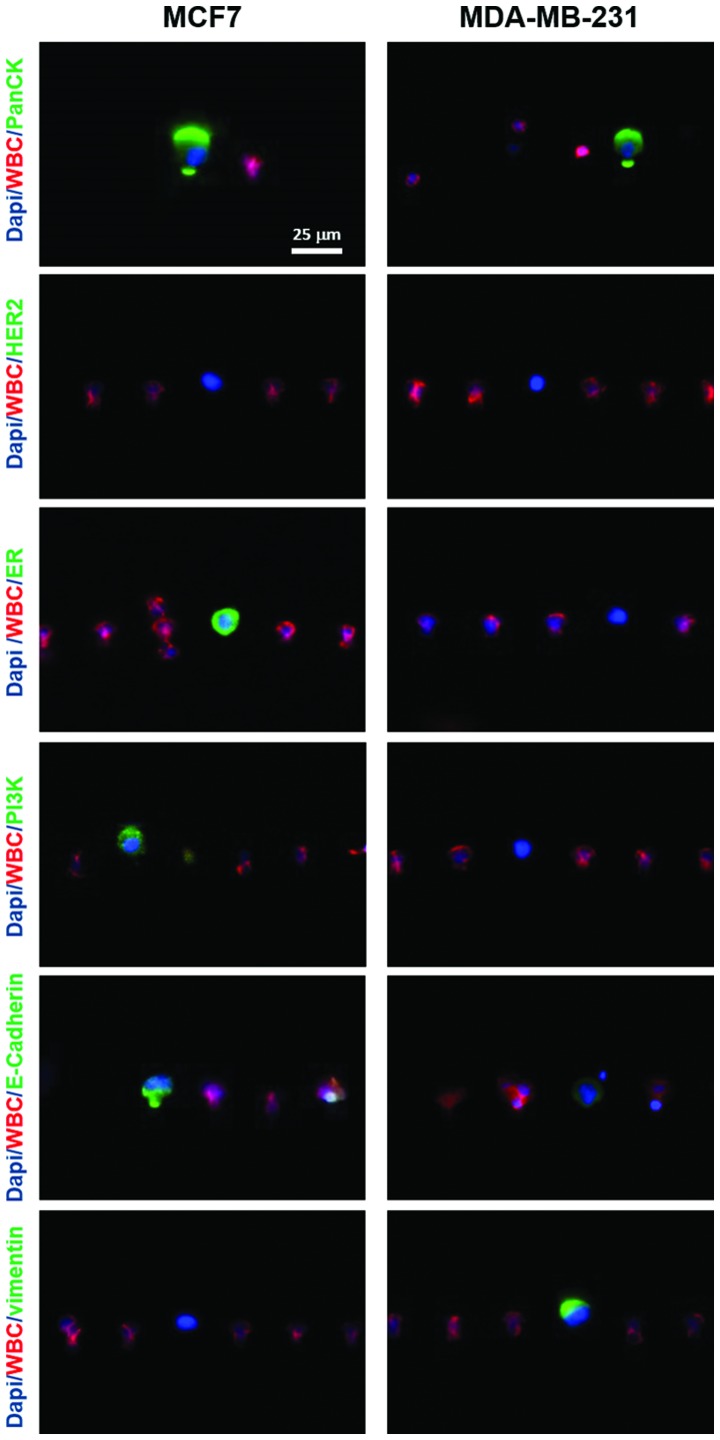
Immunostaining of captured cells spiked into human blood. For each cell line (columns), 100 cells were first spiked into peripheral blood samples from healthy donors and then stained with either PanCK, HER-2, ER, PI3K, E-cadherin or vimentin after being captured by microchambers (rows). The cell nuclei were also stained by 1.0 *μ*m/g Hoechst-33342 in all cases.

Similar analysis of the expression has been performed on the captured cells using spike-in cells into peripheral blood. To distinguish background hematologic cells from the captured cancer cells, we used CD45 as a marker for leukocyte staining. Examples of the stained captured cancer cells and leucocytes are shown in Fig. 5. The results are highly consistent with those from the cell lines. MCF7 cells were PanCK, ER, PI3K, E-cadherin positive, but HER2 and vimentin negative. MDA-MB-231 cells possessed high level of vimentin and PanCK expression and low level of E-cadherin expression, but no expression on HER2, ER and PI3K. Our results suggested that the microchannel device capture both epithelial cancer cells such as MCF7 and SKBR3 and EMT-like cells such as MDA-MB-231. Furthermore, the microchannel device is able to identify differential expression and phenotype of capture cells using panel of epithelial and mesenchymal breast cancer biomarkers. The data of the molecular characterization in spike-in cells is summarized in Table IV.

### Capture of CTCs in patient clinical samples

**Figure 6.**
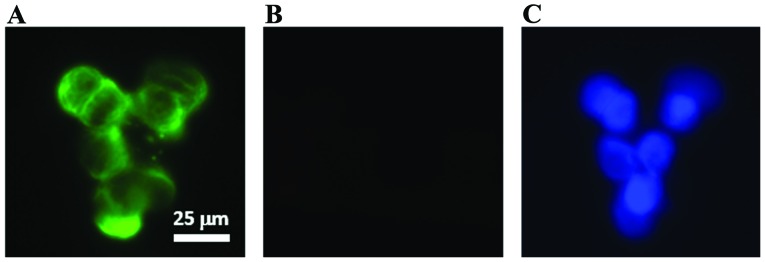
A cluster of CTCs captured using the ‘Jetta’ chip. (A) CTCs stained with Pan CK, (B) CTCs not stained with CD45, (C) CTC nuclei stained with Hoechst nuclear stain.

To test the clinical application of the microfluidic device, blood samples from metastatic breast cancer patients were processed. CTCs have been captured and enumerated using the antibodies against PanCK and CD45 (Fig. 6). From 2 ml of blood, 1 to >600 CTCs have been counted from the metastatic breast cancer samples. Interestingly, the device also captured clusters of cancer cells, which have been implicated as micrometastases and probably represent more aggressive tumor cells than individual CTCs. Detailed clinical data and further analysis of the study is being carried out with the aim towards demonstrating the clinical use of the platform.

## Discussion

We investigated a novel technology of capturing and characterizing CTCs by using a microchannel device. Different breast cancer cells including MCF7, MDA-MB-231 and SKBR3, as well as a panel of breast cancer biomarkers were used to test the device. The device can capture cells in a range of 20–2,000 with high reproducibility. The capturing efficiency of the cells is greater than 80% with a minimum background of leukocyte contamination in the captured cell population. Furthermore, it captured both epithelial cancer cells such as MCF7 and SKBR3 and mesenchymal cells such as MDA-MB-231. Immunostaining of the captured cells on the microchannel device suggested that a panel of breast cancer biomarkers can be used to characterize differential expression of the captured cells. This device is unique in its ability to segregate cancer cells in their individual chambers thus separating them from contaminating leukocytes and also allowing for on chip molecular analysis at the single cell level. This study is laying the foundation for future studies that will test the clinical validity and utility of this CTC technology.

Breast cancer represents a heterogeneous group of diseases. Cell lines derived from primary tumors can reflect the molecular diversity of the disease. One objective of this study was to investigate the expression patterns of those clinically relevant biomarkers for breast cancer (ER, HER2, PI3K, vimentin and E-cadherin) in commonly used breast cancer cells. The panel of breast cancer markers selected for the study has been implicated to be specific for breast epithelial cells and/or mesenchymal cells. The detection of the markers in the captured cells not only confirmed that the cells originated from subtypes of breast cancer, but also revealed that the majority of captured cells kept the properties of breast cancer cells. Among the three breast cancer cell lines, MCF7 resembles the Luminal A subtype because it is ER positive and HER2 negative. SKBR3 with high HER2 expression and no ER expression belongs to HER2 subtype. In contrast, MDA-MB-231 with vimentin positive, HER2 negative, and ER negative resembles within the basal-like subtype. This demonstrated the feasibility of using the biomarkers to classify different types of breast cancer cells using the microchannel platform.

We observed that CD45, a leukocyte specific marker was expressed in the majority of the background leukocytes. This level of the background leukocytes was consistent with the observation of leukocytes presence in the CTC-enriched populations with other CTC capturing technologies. Although the background leukocytes create a challenge for detecting and analyzing CTCs, the level of leukocyte background observed with this technology kept leukocytes in separate microchambers and did not seem to affect the analytical sensitivity of immunostaining of the captured cells.

In summary, clinical oncology is challenged by a lack of predictive tests for therapy choice and therapy response that are simple, non-invasive and inexpensive. CTC technologies provide a great promise of delivering such a tool that enables enumeration and molecular characterization of metastatic cancer cells and estimate prognosis and therapeutic response of the patient. Fundamental research continues to increase our knowledge of molecular and cellular processes that contribute to the clinical behavior of cancer. Further development of the technology could potentially lead to benefits of the patients through personalized treatment strategies to improve patient management and outcomes.

## Figures and Tables

**Table I.** Capturing efficiency of breast cancer cells using cell lines.

Cell line	No. of spiked cells	Capturing efficiency, %	Coefficient of variance
MCF7	50	75.0	6.7
	100	78.7	5.6
	200	82.3	5.8
	500	83.2	4.4
	1,000	80.5	3.5
	2,000	83.2	3.4
MDA-MB-231	50	77.0	6.5
	100	79.2	6.3
	200	82.8	5.2
	500	85.0	4.4
	1,000	80.5	5.6
	2,000	84.9	2.5
SKBR3	50	78.0	5.1
	100	81.2	4.2
	200	85.1	2.7
	500	89.0	4.5
	1,000	84.1	5.3
	2,000	86.9	2.6

**Table II.** Capturing efficiency of spike-in breast cancer cells in normal donor blood.

Cell line	No. of spiked cells	Capturing efficiency, %	Coefficient of variance
MCF7	50	74.0	5.6
	100	77.7	3.7
	200	79.2	3.8
	500	78.9	4.3
	1,000	79.0	4.0
	2,000	80.9	2.7
MDA-MB-231	50	75.0	6.5
	100	81.0	3.0
	200	79.0	1.0
	500	80.5	3.7
	1,000	82.6	1.5
	2,000	82.3	4.5

**Table III.** Differential expression of cancer biomarkers in breast cancer cell lines.

	MCF7	MDA-MB-231	SKBR3
PanCK	+	+	+
HER-2	−	−	+
ER	+	−	−
PI3K	+	−	+
E-cadherin	+	+ (low)	+
Vimentin	−	+	−

**Table IV.** Differential expression of cancer biomarkers in spike-in breast cancer cells.

	MCF7	MDA-MB-231
PanCK	+	+
HER-2	−	−
ER	+	−
PI3K	+	−
E-cadherin	+	+ (low)
Vimentin	−	+
